# Deep learning-based reconstruction and superresolution for MR-guided thermal ablation of malignant liver lesions

**DOI:** 10.1186/s40644-025-00869-x

**Published:** 2025-04-02

**Authors:** Moritz T. Winkelmann, Jens Kübler, Sebastian Gassenmaier, Dominik M. Nickel, Antonia Ashkar, Konstantin Nikolaou, Saif Afat, Rüdiger Hoffmann

**Affiliations:** 1 Department of Diagnostic and Interventional Radiology, University Hospital of Tübingen, Hoppe-Seyler-Straße 3, 72076 Tübingen, Germany; 2 Cluster of Excellence iFIT (EXC2180), Eberhard-Karls University, Tübingen, Germany; 3 MR Application Predevelopment, Siemens Healthcare AG, Forchheim, Germany

## Abstract

**Objective:**

This study evaluates the impact of deep learning-enhanced T1-weighted VIBE sequences (DL-VIBE) on image quality and procedural parameters during MR-guided thermoablation of liver malignancies, compared to standard VIBE (SD-VIBE).

**Methods:**

Between September 2021 and February 2023, 34 patients (mean age: 65.4 years; 13 women) underwent MR-guided microwave ablation on a 1.5 T scanner. Intraprocedural SD-VIBE sequences were retrospectively processed with a deep learning algorithm (DL-VIBE) to reduce noise and enhance sharpness. Two interventional radiologists independently assessed image quality, noise, artifacts, sharpness, diagnostic confidence, and procedural parameters using a 5-point Likert scale. Interrater agreement was analyzed, and noise maps were created to assess signal-to-noise ratio improvements.

**Results:**

DL-VIBE significantly improved image quality, reduced artifacts and noise, and enhanced sharpness of liver contours and portal vein branches compared to SD-VIBE (*p* < 0.01). Procedural metrics, including needle tip detectability, confidence in needle positioning, and ablation zone assessment, were significantly better with DL-VIBE (*p* < 0.01). Interrater agreement was high (Cohen κ = 0.86). Reconstruction times for DL-VIBE were 3 s for k-space reconstruction and 1 s for superresolution processing. Simulated acquisition modifications reduced breath-hold duration by approximately 2 s.

**Conclusion:**

DL-VIBE enhances image quality during MR-guided thermal ablation while improving efficiency through reduced processing and acquisition times.

## Introduction

Image guidance using magnetic resonance has shown considerable promise in various applications in interventional radiology. Thermal ablation, including radiofrequency and microwave ablation, is a widely used minimally invasive treatment modality for liver tumors, mainly under CT or ultrasound guidance due to accessibility, real-time imaging capabilities, and established clinical workflows [1, 2]. Unlike CT or ultrasound, MRI for thermal ablation of liver tumors allows visualization of tumors and surrounding anatomy without contrast medium, provides real-time fluoroscopy from multiple angles, and facilitates simultaneous monitoring of the applicator and tumor, even in difficult positions [3]. MRI enables precise targeting, continuous monitoring, and immediate assessment of success by utilizing different signal variations between coagulated and non-coagulated tissue [4, 5]. Although MRI offers these advantages, it is not yet widely established for the thermal ablation of liver tumors. Limited adoption is due to the complexity of procedures, high costs, and lengthy durations, restricting them to specialized centers [6, 7].

Challenges with MR-guided interventions include prolonged image acquisition times compared to other modalities and suboptimal visibility of the applicator tip, which may lead to inaccuracies in estimating its length and exact position relative to the tumor and critical structures depending on the device and sequence [7, 8]. Additionally, motion artifacts, inadequate breath-holding, and smaller coils, can further compromise image quality during these procedures [3, 7].

Recently, deep learning-accelerated sequences and post-processing algorithms have been developed to enable fast data acquisition and improved image quality [9, 10]. These methods have demonstrated effectiveness in reconstructing images across a variety of contrasts and anatomical regions in MRI, including but not limited to the abdomen, brain, musculoskeletal system, genitourinary and gastrointestinal tracts, reducing examination time and improving image resolution [9, 11–15]. During MR-guided microwave ablation, the T1-weighted volume-interpolated breath-hold examination (VIBE) sequence is acquired multiple times to determine the exact positioning of the tumor, verify the placement of the microwave applicator in the path of the tumor lesion, and assess the success of the ablation by evaluating the T1w hyperintense ablation zone [7].

A recent study demonstrated the clinical utility of a deep learning-based super-resolution technique for VIBE sequences in pre- and post-contrast abdominal MRI [16]. This method, developed by training a neural network on high-resolution head and pelvis data, improves image quality by reducing noise, sharpening edges, increasing clarity and reducing Gibbs ringing. Furthermore, recent studies have shown additional benefits when deep learning-based image enhancement is integrated into the image reconstruction from k-space data [17, 18].

The aim of this study was to investigate the feasibility of the deep learning-based reconstruction and superresolution techniques (DL-VIBE) for standard T1-weighted VIBE-MRI (SD-VIBE) acquired during MR-guided thermal ablation, particularly in terms of speed of image acquisition and reconstruction, as well as potentially improved diagnostic image quality and greater diagnostic confidence during treatment monitoring.

## Methods

### Study design and patient characteristics

Between September 2021 and February 2023, a total of 34 consecutive patients who underwent clinically indicated MRI-guided thermal ablation of a malignant liver tumor were included. The T1-weighted 3D VIBE datasets, acquired during the intervention, were retrospectively processed using a two-step deep learning-based image reconstruction. All data sets were acquired using non-contrast VIBE sequences and standardized imaging parameters, with a consistently positioned and uniform coil type, ensuring reproducibility while allowing for minor positional adjustments based on procedural requirements. This retrospective single center study was approved by the institutional review board (*BLINDED*).

### Interventional procedure and imaging protocol

All ablations were performed using a high-power microwave ablation system with a maximum generator output of 150 W and an operating frequency of 2.45 GHz, with each procedure conducted by one of the four radiologists from the MRI-guided thermal ablation team. The system includes a perfusion pump to cool the applicator shaft. A 14-G MR-compatible microwave applicator (ECO-100AI13C, Nanjing ECO Medical Instrument Co., China) with a length of 15 cm was used for the procedures. The applicator features a titanium alloy shaft and a ceramic tip. The microwave generator (ECO-100E2, Nanjing ECO Medical Instrument Co., China) was placed outside the scanner room during the procedures, connected to the MR-compatible antenna via a 4-meter coaxial cable.

The procedures took place in a wide-bore 1.5-T system (Siemens MAGNETOM Aera, Siemens Healthineers, Erlangen, Germany) designed for MR-guided interventions. A radiofrequency-shielded liquid crystal display monitor was positioned next to the scanner’s opening, allowing real-time monitoring during the procedure, with the interventionalist seated close to the patient lying inside the scanner.

The entire procedure, including planning imaging, tumor targeting, therapy monitoring, and control imaging, was performed with the patient positioned on the scanner table. Analgesia (*n* = 27) or general anesthesia (*n* = 7) was administered during the procedures. In patients receiving analgesia, respiratory motion was actively managed by the interventionalist through standardized breath-hold commands, specifically during needle puncture and advancement.

In patients under general anesthesia, controlled ventilation was administered by the attending anesthesiologist within the MR suite. The potential puncture site on the skin was marked using a capsule (Nifedipine AL 5, Aliud Pharma, Laichingen, Germany), followed by the acquisition of unenhanced planning sequences. After disinfection, a six-channel body array coil was positioned at the puncture site, allowing the applicator to be inserted through one of the four openings in the coil. Following sterile draping and local subcutaneous anesthesia (Xylocaine 1%, AstraZeneca, Wedel, Germany), a small incision was made at the entry point. A 3D T1-weighted VIBE sequence was acquired with the microwave applicator placed in the subcutaneous tissue. This sequence was utilized to adjust the imaging slices for an MR fluoroscopic sequence (BEAT-Multislice), enabling near-real-time tracking of the applicator in three imaging planes during tumor targeting. The 3D T1-weighted VIBE sequence was repeated to confirm the correct positioning of the applicator. Due to previously reported issues with applicator tip visibility, extra caution was taken during the placement and position verification of the applicator [8]. Ablation was initiated after connecting the microwave antenna to the generator via an MR-compatible coaxial cable, with the generator situated outside the scanner room. After the ablation, an unenhanced 3D T1-weighted VIBE sequence was acquired for therapy monitoring without removing the microwave antenna. If the ablation zone, identified as a hyperintense area in T1-weighted imaging [19], was deemed insufficient, additional ablation was performed either with the applicator in the same position or after repositioning it. If the ablation zone was deemed adequate, covering the target tumor with a sufficient safety margin of over 5 mm, the applicator was retracted under coagulation.

Post-procedural control imaging, including an axial T2-weighted TSE sequence and multiphasic contrast-enhanced 3D T1-weighted Dixon VIBE after intravenous administration of gadobutrol at 0.1 mmol/kg body weight (Gadovist, Bayer HealthCare), was performed to assess technical success and rule out complications such as bleeding.

All intraprocedural T1-VIBE sequences were performed with an echo time of 1.3 ms, repetition time of 3.5 ms, slice thickness of 2 mm, matrix size of 320 × 189, flip angle of 10°, and a bandwidth of 400 Hz/pixel. Following the clinical procedure, the raw data from all T1-VIBE sequences (median: 22.5 sequences per patient; interquartile range: 15.8–31.3) were exported from the MR scanner for post-processing.

### Deep learning-based image reconstruction and retrospective processing

The employed deep learning-based image reconstruction comprised two sequential steps. First, images were reconstructed from k-space data with the acquired resolution using an architecture inspired by variational networks [20] using the same algorithm as recently reported for clinical investigations in abdominal imaging [17, 18]. The algorithm performs six iterations that alternate between parallel imaging-based data consistency and neural network-based image enhancement. Its trainable parameters consist of network parameters as well as step-sizes for the data consistency update and were determined through supervised training using about 500 fully sampled datasets obtained from healthy volunteers in various body regions. These processing steps have been described in detail in several studies [17, 18]. Second, the obtained images were interpolated in all spatial directions using the deep learning-based superresolution algorithm that was furthermore tailored to partial Fourier acquisitions that was recently explored and detailed in several studies [13, 16]. The approach is motivated by the observation that conventional algorithms for partial Fourier processing with assumptions on the background phase are not suited for gradient-echo sequences but can be replaced using neural networks. Both steps were integrated into a research application that was provided by Siemens Healthineers as a research-software-package for prospective use on the scanner.

### Qualitative image analysis, evaluation of intervention-related parameters and quantitative noise maps

Two experienced interventional radiologists, each with 6 and 7 years of experience and both integral members of the MRI-guided thermal ablation team, independently evaluated the image datasets. Each reader had access to the complete imaging dataset for each patient, including all sequences acquired throughout the procedure in the transverse plane, with additional coronal or sagittal sequences available when applicable. The datasets were randomized and blinded to patient identifiers, procedural details, and chronological order to minimize bias. The radiologists independently reviewed and compared deep learning-enhanced VIBE (DL-VIBE) sequences with standard unenhanced VIBE (SD-VIBE) sequences. They assessed diagnostic confidence in lesion detection, overall image quality, the presence of noise and artifacts, sharpness of the liver and portal vein branches and the visbility of the costodiaphragmatic recess, which is critical to avoid direct contact with the applicator and reduce the risk of pneumothorax. They also assessed the contrast between the ribs and intercostal spaces, which aids in preventing inadvertent puncture of intercostal arteriesVisibility of the needle artifact in the liver, and confidence in the positioning of the needle in relation to the presumed trajectory to the lesion were assessed, which may have a direct impact on the accuracy of the ablation procedure. Needle positioning was assessed by evaluating its alignment with the presumed trajectory to the lesion, while ablation zone adequacy was determined by assessing whether the entire lesion was contained within the ablation area with an appropriate safety margin. Although no formal measurements of the safety margin were performed during image review, readers subjectively confirmed that the entire lesion was included within the ablation zone with an estimated margin of at least 5 mm, based on visual assessment.

Each of these parameters was assessed using a 5-point Likert scale, with 1 indicating poor, 2 fair, 3 moderate, 4 good, and 5 excellent. Noise and artifacts were evaluated using a separate scale, with 1 indicating major, 2 substantial, 3 moderate, 4 minor, and 5 none. To illustrate the effect of the deep learning-based image enhancement, exemplary signal-to-noise (SNR) maps were calculated using the pseudo-replica method [21].

### Statistical analysis

The available data were analyzed using SPSS (SPSS Statistics 29, IBM Corp., Armonk, New York, NY, USA). Continuous variables are presented as the mean ± standard deviation. The Shapiro-Wilk test was used to assess normality of the data. As the data were not normally distributed, comparisons between groups were made using non-parametric methodology (Wilcoxon test). Intraclass correlation (ICC) was used to calculate the interrater agreement. ICC values ≤ 0.5 were defined as poor, those 0.51–0.75 were defined as moderate, those 0.76–0.90 were defined as good, and those > 0.90 were defined as excellent consistency. A *p*-value < 0.05 was considered statistically significant.

## Results

A total of 34 consecutive patients with malignant liver tumors (mean age: 65.4 ± 11.5, 13 women, 21 men) with complete MRI data sets were included. All interventions were technically successful, with a median intervention time of 122.5 min (range: 75–197 min). The different tumor entities of the liver lesions for the included patients are listed in Table 1. The 3D-VIBE sequence was successfully reconstructed in all patients, with a median of 22.5 sequences per patient and an interquartile range of 15.8–31.3 sequences.

**Table 1:** Baseline patient characteristics

Baseline characteristics	*N* (%)/mean ± SD
Age (years)	65.4 ± 11.5
Patients	34
Sex (m/f)	21 (62%) / 13 (38%)
Number of liver lesions	42
Tumor size of ablated lesions	15.7 ± 8.6 mm (range: 5–44 mm)
Tumor entity	Colorectal cancer (*n* = 12)
	Hepatocellular carcinoma (*n* = 9)
	Neuroendocrine tumor (*n* = 6)
	Melanoma (*n* = 4)
	Squamous cell carcinoma (*n* = 1)
	Cholangiocarcinoma (*n* = 1)
	Gastroesophageal junction adenocarcinoma (*n* = 1)

### Image quality and interventional related parameters

**Fig. 1:**
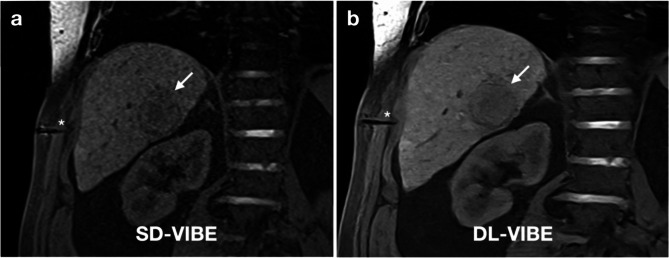
62-year old male patient with hepatocellular carcinoma in the right lobe of the liver. The SD-VIBE sequence (**a**) shows considerable artifacts and noise as well as a difficult delineation of the needle artifact of the microwave antenna (asterisk) in the subcutaneous fatty tissue. With DL-VIBE (**b**), the needle tip artifact is much easier to differentiate, as is the presumed trajectory, with improved sharpness of the peripheral portal vein branches, the liver parenchyma and the target lesion (arrow)

The general image quality and sharpness of liver contrast were significantly higher with DL-VIBE (median 4, interquartile range 2) compared to SD-VIBE (3, IQR 2) (*p* < 0.01). Additionally, noise was significantly lower with DL-VIBE (5, IQR 2) compared to SD-VIBE (3, IQR 2), and artifacts were also reduced with DL-VIBE (4, IQR 2) compared to SD-VIBE (3, IQR 2) (both *p* < 0.01). Overall image sharpness was significantly higher with DL-VIBE (5, IQR 2) compared to SD-VIBE (3.5, IQR 2) (*p* < 0.01). The visibility of procedure-specific organic structures was significantly enhanced with DL-VIBE compared to SD-VIBE. Specifically, the contrast of the costodiaphragmatic recess was higher with DL-VIBE (5, IQR 2) compared to SD-VIBE (4, IQR 2), the contrast between ribs and soft tissue improved with DL-VIBE (4, IQR 2) versus SD-VIBE (3, IQR 2), and the sharpness of second-order portal vein branches was significantly better with DL-VIBE (5, IQR 3) compared to SD-VIBE (3.5, IQR 1) (all *p* < 0.01). The detection of the target lesion was better with DL-VIBE (4, IQR 2) compared to SD-VIBE (4, IQR 1) (*p* < 0.01) (Fig. 1). Moreover, the visibility of needle artifacts in the subcutaneous fatty tissue and liver parenchyma was significantly improved with DL-VIBE (5, IQR 3) compared to SD-VIBE (3, IQR 2) (*p* < 0.01) (Fig. 1).

**Fig. 2:**
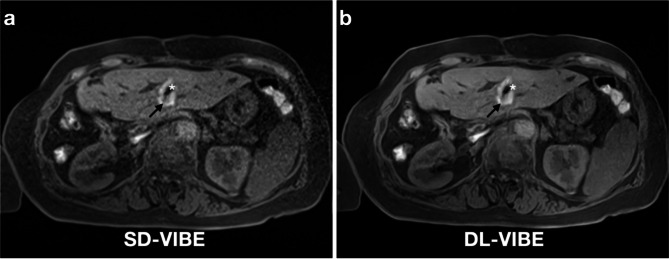
58-year old female patient with colorectal metastasis in liver segment 3. The T1 weighted hyperintense ablation zone around the target lesion (arrow) and the artifact of the needle tip (asterisk) are present. While clear noise can still be seen in the SD-VIBE sequence (**a**), the ablation zone and the needle tip can be assessed significantly better with the DL-VIBE sequence (**b**), which is crucial for the evaluation of the technical success of the procedure

**Fig. 3:**
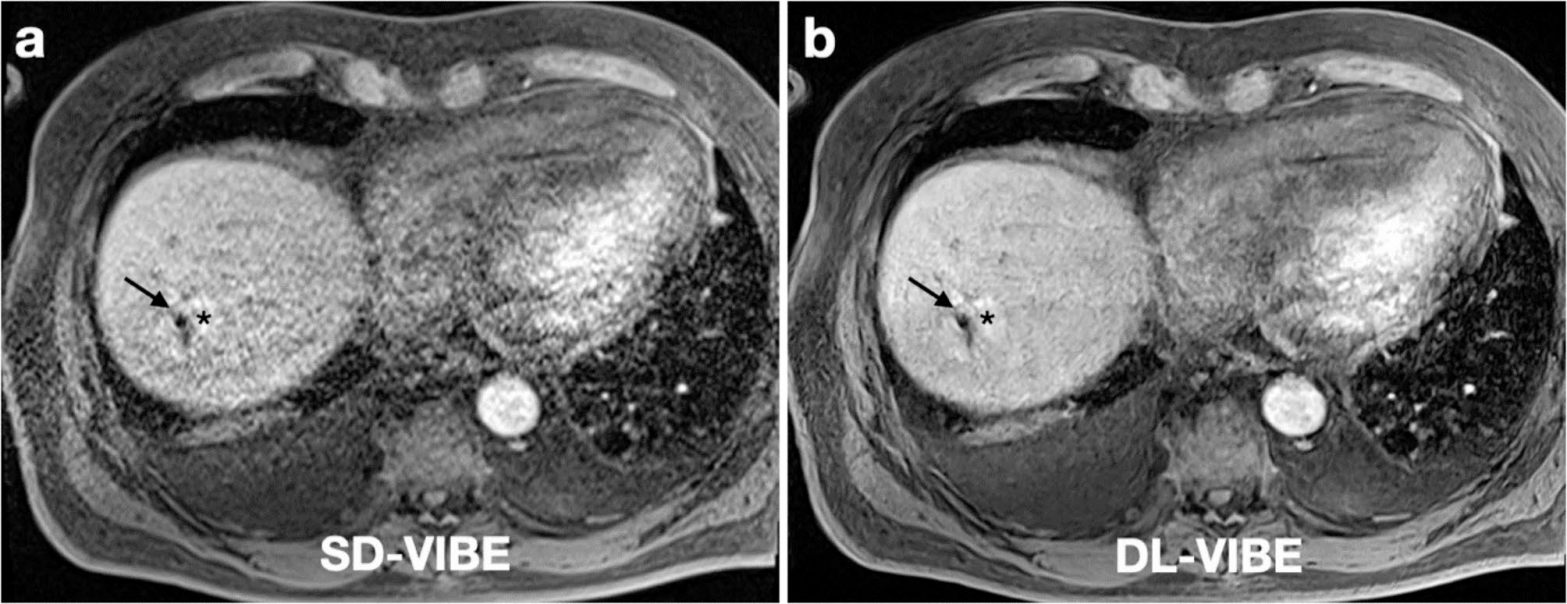
54-year-old male patient with a liver metastasis of colorectal carcinoma in the right hepatic lobe after microwave ablation with the antenna tip still in place (arrow). The SD-VIBE sequence (**a**) shows considerable motion artifacts and a difficult demarcation of the ablation zone (asterisk). With DL-VIBE (**b**), the artifacts are significantly reduced and the extent of the ablation zone is easier to assess (asteriks)

Confidence in key procedural aspects was significantly higher with DL-VIBE compared to SD-VIBE. The detectability of the intrahepatic antenna tip was improved with DL-VIBE (5, IQR 1) compared to SD-VIBE (4, IQR 2). Similarly, confidence in the needle position within the liver was higher with DL-VIBE (5, IQR 1) versus SD-VIBE (4, IQR 2). The correct positioning of the antenna in the lesion was assessed with greater certainty using DL-VIBE (5, IQR 1) compared to SD-VIBE (4, IQR 2). Lastly, diagnostic confidence in the ablation zone was significantly higher with DL-VIBE (5, IQR 1) than with SD-VIBE (4, IQR 2) (all *p* < 0.01) (Figs. 2 and 3).

Table 2 shows the ratings of the two readers for all parameters in detail.

Interrater agreement across these evaluations was high, as indicated by a Cohen κ value of 0.86 (CI: 0.84, 0.88), demonstrating strong consistency among the raters.

**Table 2:** Evaluation of image quality criteria and intervention-related parameters between standard VIBE datasets and VIBE datasets with deep learning-based reconstruction and superresolution. All parameters were assessed in all patients (*N* = 34)

Parameters	SD-VIBE (Reader 1/Reader 2)	DL-VIBE (Reader 1/Reader 2)	*p*-value
**Image quality**			
Overall image quality	3 (2)	4 (2)	< 0.01
Noise	3 (2)	5 (2)	< 0.01
Artifacts	3 (2)	4 (2)	< 0.01
Sharpness	3.5 (2)	5 (2)	< 0.01
**Visbility of anatomic structures**			
Contrast of costodiaphragmatic recess	4 (2)	5 (2)	< 0.01
Contrast of ribs to soft tissue	3 (2)	4 (2)	< 0.01
Sharpness of second order portal vein branches	3.5 (1)	5 (3)	< 0.01
**Procedure-related parameters**			
Target lesion detection	4 (1)	4 (2)	< 0.01
Subcutaneous needle artifact detectability	3 (2)	5 (3)	< 0.01
Detectability of the intrahepatic antenna tip	4 (2)	5 (1)	< 0.01
Confidence in needle position in the liver	4 (2)	5 (1)	< 0.01
Confidence in the correct position of the antenna in the lesion	4 (2)	5 (1)	< 0.01
Diagnostic confidence in ablation zone	4 (2)	5 (1)	< 0.01

Data are given as median (interquartile range)

SD-VIBE: standard volume-interpolated breath-hold examination

DL-VIBE: deep learning-enhanced volume-interpolated breath-hold examination

### Reconstruction and acquisition time

The reconstruction time for the acquisition protocols employed in this study was determined in a phantom experiment. Utilizing a scanner integrated GPU, both protocols employed in this study required about 3 s for the first reconstruction step and 1 s for the following superresolution algorithm. The retrospective reconstructions furthermore simulated a shorter acquisition time by discarding some of the acquired phase encoding steps. While the parallel imaging acceleration was not modified, the k-space coverage was reduced to correspond to a partial Fourier acquisition with a partial Fourier factor of 6/8 in both phase encoding directions. In a prospective acquisition this would translate to a scan time reduction from 13.9 s to 11.9 s for the axial acquisition and from 13.0 s to 11.2 s for the coronal acquisition. The retrospective reconstructions were performed using a research application that allows reprocessing in the scanner environment and performing retrospective undersampling. Therefore, all evaluated images with retrospective reconstruction require a reduced acquisition time of about 2 s.

### SNR maps

**Fig. 4:**
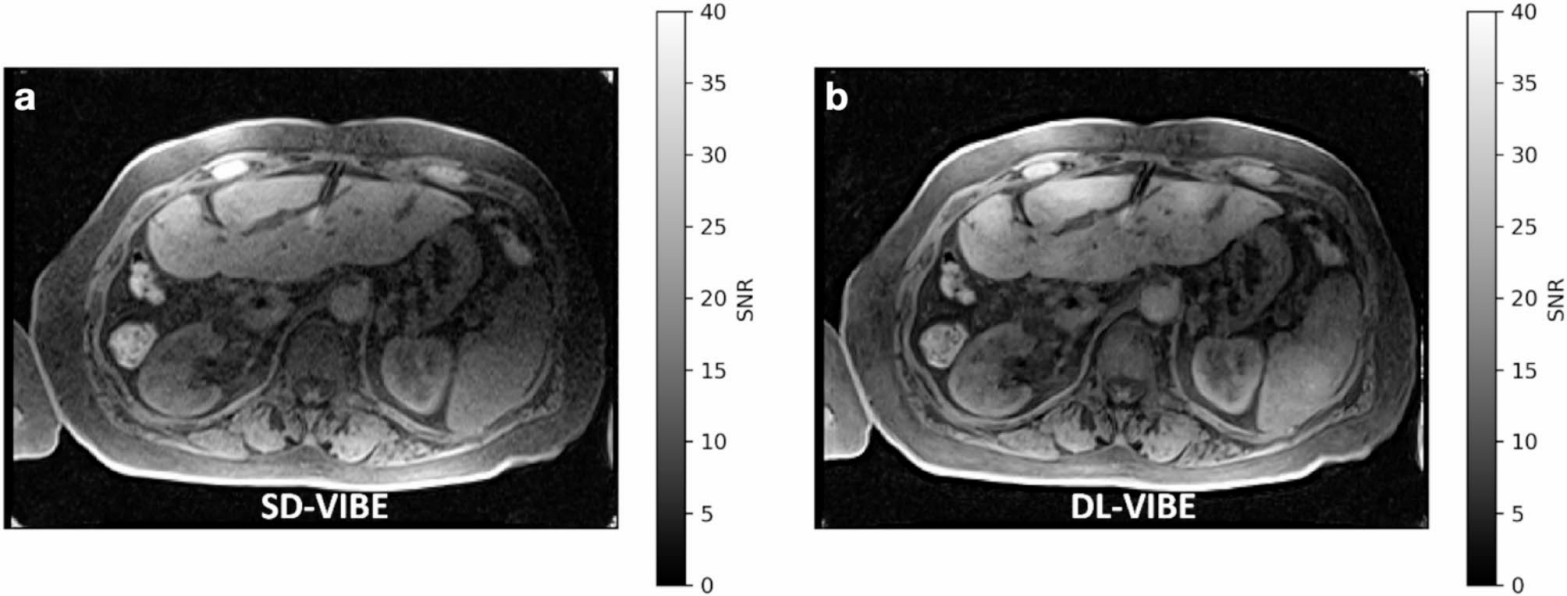
Exemplary SNR map for SD-VIBE (**a**) and DL-VIBE (**b**) processing. The DL-VIBE shows slightly increased SNR even though the reconstruction process uses fewer data samples and involves a superresolution component

For an exemplary dataset, SNR maps were estimated using the pseudo-replica method [21] with a slice illustrated in Fig. 4. The SNR map of the conventional reconstruction appears like an unnormalized image. This is expected as the spatial variation of noise is only modified by the parallel imaging reconstruction and moderate for low parallel imaging acceleration as chosen in the interventional protocols. The SNR of the DL reconstruction shows slightly more variation, improving in homogeneous tissue. Even though the DL reconstruction uses fewer data for the reconstruction and involves superresolution as an interpolation step that usually enhances noise and reduces SNR, the obtained SNR is at least on the level of the conventional reconstruction.

## Discussion

In our study, we investigated the effectiveness of a novel deep learning-based reconstruction method to improve the sharpness and noise reduction of non-contrast T1-weighted 3D VIBE images during MR-guided thermal ablation. Our results show that this approach enables the acquisition of images characterized by improved delineation of organ boundaries, increased sharpness and reduced artefacts. Similar results have already been demonstrated with this technique for the T1-weighted volume-interpolated breath-hold examination in diagnostic magnetic resonance imaging of the upper abdomen [16].

The application of this technique also led to significant improvements in critical procedural parameters for thermal ablation. Improved visualization of the subcutaneous needle artifact at the start of the procedure and improved clarity of the costodiaphragmatic recess were achieved. These improvements are particularly important for accurately assessing the position of the tip of the antenna in relation to the lung, reducing the risk of accidental lung puncture and subsequent pneumothorax [22]. In addition, the increased rib contrast enabled better visibility of the needle tip in relation to the lower edge of the rib, which is of particular relevance in intercostal access [23]. In addition, our method significantly improved the visibility of needle artifacts within the liver, which increased the accuracy of needle placement in relation to the intrahepatic portal vein branches, which is essential for precise and safe targeting during ablation [7]. The DL method also increased confidence in the positioning of the needle along the presumed trajectory to the lesion, as assessed by comparing its alignment across multiple images acquired in different planes. This is important when the target tumor is located in front of critical structures and a misrepresentation of the antenna tip can lead to an accidental puncture of the structure [24]. In MRI, the occurrence of applicator artifacts is usually determined by the material and size of the applicator, the differences in magnetic susceptibility between the applicator and the surrounding tissue and can also be influenced by the angle to the primary magnetic field [25, 26].

In addition, target lesion detection and assessment of ablation zone adequacy were improved with DL-VIBE, increasing diagnostic certainty regarding whether ablation included the target lesion at the end of the procedure. This analysis was also based on non-contrast-enhanced T1-weighted VIBE sequences, which utilized the ability of MRI to distinguish coagulated from uncoagulated tissue without the need for contrast agent administration [3, 7].

These results emphasize the clinical relevance of the proposed technique and highlight its potential to significantly improve the safety and precision of MRI-guided thermal ablation procedures.

We also created a sample noise map in our study to illustrate the improvement in SNR, and although the super-resolution method used achieves higher resolution, this often leads to a further reduction in SNR; high-resolution images may allow better delineation of smaller areas but also increase scan times, with overall image quality and high spatial resolution being the result of a complex interplay between image quality, resolution, SNR and scan time [27]. However, it is crucial to minimize scan times, especially for time-intensive procedures such as MRI-guided thermal ablation and for the sensible allocation of resources. One possible approach to solving this problem is the use of reconstruction methods, such as the one investigated in this study. The DL-VIBE method, a research application based on deep learning for reconstruction super-resolution, interpolates low-resolution images to produce high-resolution results through noise reduction, edge enhancement and Gibbs ring reduction. In contrast to traditional polynomial interpolation, this method uses deep learning, which is data-driven and applies prior knowledge from large datasets to improve MR images by denoising and deblurring lower resolution scans [28, 29]. In addition to improving image quality and evaluating procedure-specific parameters, DL sequences have led to time savings in image acquisition and reconstruction. Although the time savings per sequence are minimal in relation to the total duration of the procedure, on average 146.4 ± 26.2 min, as observed in an earlier study on MR-guided microwave ablation [30], these reductions are clinically highly relevant. Shorter breath-holding maneuvers improve respiratory compliance during shorter sequences, which is beneficial, particularly for patients undergoing procedures without general anesthesia, enhancing their comfort. Furthermore, a significant portion of the procedure time is often dedicated to planning and control imaging, during which the interventionalist is outside the scanner room, and the microwave applicator is not yet or no longer inserted into the patient. The 3D VIBE sequence is primarily for targeting and monitoring, with the interventionalist in sterile clothing spending most of the time holding and positioning the applicator while the patient remains in the MRI bore. This scenario can be uncomfortable for the patient, especially if not under general anaesthesia, and challenging for the interventionalist with or without anaesthesia. Therefore, any time saved is beneficial for both the patient and the interventionalist.

Our study has some limitations, one of which is the small number of patients. In addition, the 3D VIBE sequences were retrospectively processed and not used during the actual interventions. The image quality assessments were performed retrospectively and may be assessed differently by other interventionalists during the actual procedures. Due to the retrospective design, the time saving is also only theoretical and it is unclear whether this is actually feasible during an intervention. To obtain a more meaningful result regarding the benefits of DL-VIBE, it should be installed on the device and integrated into the protocol, and a new assessment should be performed regarding actual image quality improvements and time savings.

The implementation of DL-VIBE improves image quality and time efficiency in MR-guided thermal ablation procedures. While the integration of deep learning technologies shows promise in enhancing procedural guidance and assessment, its impact on diagnostic reliability and procedural accuracy requires further prospective investigation.

## Data Availability

Raw data were generated at University Hospital Tuebingen, Department of Diagnostic and Interventional Radiology. Derived data supporting the findings of this study are available from the corresponding author M.T.W. on request.

## Abbreviations

DL-VIBE: Deep Learning-enhanced Volume Interpolated Breath-hold Examination
GPU: Graphics Processing Unit
ICC: Intraclass Correlation Coefficient
SD-VIBE: Standard Volume Interpolated Breath-hole Examination
SNR: Signal-to-Noise Ratio
TSE: Turbo Spin Echo
